# Immobilized nanoscale zero-valent iron for synergistic enhanced removal of pentachlorobenzene with *Pseudomonas* sp. JS100

**DOI:** 10.3389/fbioe.2022.1089212

**Published:** 2022-11-30

**Authors:** Anzhou Ma, Jianpeng Ma, Xianke Chen, Guoqiang Zhuang

**Affiliations:** ^1^ Research Center for Eco-Environmental Sciences, Chinese Academy of Sciences, Beijing, China; ^2^ College of Resources and Environment, University of Chinese Academy of Sciences, Beijing, China

**Keywords:** PeCB, nanoscale zero-valent iron, biofilter, *Pseudomonas* sp. JS100, synergistic degradation

## Abstract

Highly chlorinated benzenes usually have a low efficient degradation in environment. Here we proposed a synergistic removal strategy of pentachlorobenzene (PeCB) using *Pseudomonas* sp. JS100 coupled with immobilized nanoscale zero-valent iron (NZVI). The structural and textural features of the synergistic system were characterized by X-ray powder diffraction, field emission scanning electron microscopy, and a specific surface area and pore size analysis. Nanoscale zero-valent iron particles were dispersed and attached to the biofilter, which increased the specific surface area to 34.5 m^2^ g^−1^. The batch experiment revealed that the removal efficiency of PeCB reached 80.2% in the synergistic system within 48 h. The degradation followed pseudo-first-order reaction kinetics, and the reaction rate constant was measured to be 0.0336 h^−1^. In the degradation mechanism, PeCB was degraded by NZVI to lower chlorobenzenes, which were utilized by *Pseudomonas* sp. JS100 as nutrients, thereby achieving rapid removal of PeCB.

## 1 Introduction

Chlorinated benzenes (CBs) are a group of substituted benzene compounds with only chlorine atoms and hydrogen atoms in the benzene ring that are widely used as biocides, degreasers, fire retardants, and chemical intermediates for the production of several other compounds, such as dyes and pharmaceutical products (Brahushi et al., 2017). Because of their high toxicity, environmental persistence, accumulation in the food chain, and migration in the water environment, CBs pose potential threats to human health and ecological security worldwide (Zhang and Wu, 2017).

Biodegradation is considered to be the main process of CBs removal in soil (Barber et al., 2005). Dechlorination is the key reaction involved in the degradation of CBs (Brahushi et al., 2004), and oxidative dichlorination and reductive dechlorination are the most prevalent biological dechlorination mechanisms in nature. The capacity of microorganisms to efficiently mineralize these toxic compounds has only been demonstrated for benzenes with four or fewer atoms of chlorine (De Bont et al., 1986; Field and Sierra-Alvarez, 2008). In contrast, similar approaches failed to achieve any significant biodegradation of pentachlorobenzene (PeCB) and hexachlorobenzene (HCB). For example, the strain *Pseudomonas* sp. JS100 has the ability to degradation of trichlorobenzene but not that with higher atoms of chlorine. Reductive dechlorination is the only known biodegradation mechanism for highly chlorinated benzenes. After reductive dechlorination under anaerobic conditions, the resulting lower chlorinated degradation products are more easily available for biomineralization. However, there are some disadvantages to reductive dechlorination such as a long remediation time and low removal efficiency, which limit the efficient degradation of PeCB and HCB (Zhou et al., 2012; Zhou et al., 2013).

In recent years, nanoscale zero-valent iron (NZVI) has been successfully employed for the removal of chlorinated contaminants. NZVI particles possess a significant surface area to weight ratio, which leads to a large density of reactive sites and contaminant removal capacity (Sarkar et al., 2012; Ghasemzadeh et al., 2014; Wang et al., 2014). Although NZVI has been widely used in water treatment technology, there are still many problems. For example, the rapid deposition caused by NZVI aggregation reduces the catalytic activity and degradation capacity (Sun et al., 2007; Gunawardana et al., 2011; Fang et al., 2022). Additionally, during the degradation of chlorinated contaminants, NZVI also adsorbs other organic matters, resulting in the consumption of active sites and reduced reaction efficiency (Zhang et al., 2011). Potential method combined the dehalogenation of NZVI with the mineralization of microorganisms was mentioned (He et al., 2009), however, there are still many challenges to its application for the toxicity of NZVI to microorganisms and the aggregated inactivation of NZVI (Xie et al., 2017).

Thus, the present study was conducted to construct a synergistic system containing *Pseudomonas* sp. JS100, NZVI, and a biofilter (JS100/NZVI/BF) for the degradation of CBs pollutants. Biofilters are used to create a microenvironment that is conducive to the dispersion of NZVI and the survival of microorganisms to enhance the degradation efficiency of CBs pollutants. In this study, the degradation ability of JS100/NZVI/BF was tested, and its degradation mechanism was explored.

## 2 Materials and methods

### 2.1 Materials and chemicals

Potassium dihydrogen phosphate (K_2_HPO_4_), magnesium sulfate hydrate (MgSO_4_·7H_2_O), ammonium nitrate (NH_4_NO_3_), sodium chloride (NaCl), ferrous sulfate hydrate (FeSO_4_·7H_2_O), zinc sulfate hydrate (ZnSO_4_·7H_2_O), manganese sulfate hydrate (MnSO_4_·H_2_O), and anhydrous ethanol were purchased from Sinopharm Group Chemical Reagent Co. Ltd. (Shanghai, China). PeCB and potassium borohydride (KBH_4_) were purchased from Shanghai Aladdin Biochemical Technology Co. Ltd. (Shanghai, China). Methanol (99.9%) and *n*-hexane (99.9%) were purchased from Thermo Fisher Scientific (China) Co. Ltd. 5,5-dimethyl-1-pyrrolidine-*N*-oxide (DMPO) and dimethyl sulfoxide (DMSO) were purchased from Sigma Aldrich. All chemicals were analytical grade, and solvents were of HPLC grade. The microorganism, *Pseudomonas* sp. JS100 [ATCC 700442], was obtained from the American Type Culture Collection (ATCC). The culture medium, Tryptic Soy Broth [BD 211825], was purchased from Becton, Dickinson and Company (BD) to recover *Pseudomonas* sp. JS100. Deionized water was used for all experiments.

### 2.2 Synthesis and characterization of NZVI and NZVI/BF

NZVI/BF was synthesized by a carrier precipitation method under nitrogen protection using the method described by Chen et al. (2018). Briefly, FeCl_3_·6H_2_O was dissolved in a three-neck flask with deionized water and ethanol. Biofilters, prepared in this study, were put into the FeCl_3_ solution, after which KBH_4_ was added into the flask to generate NZVI in the surficial pores of the biofilters. The composition and crystalline structure of NZVI/BF were then characterized by X-ray diffraction (XRD) using a PANalytical X’pert Pro X-ray diffractometer. The specific surface area was estimated by the Brunauer-Emmett-Teller (BET) method, while the external surface area, micropore surface area, and micropore volume were calculated using the t-plot method of De Boer. The surface morphology of NZVI/BF was observed with a HITACHI SU-8020 field emission scanning electron microscope (FE-SEM) after a thin film of gold was sprayed onto the samples.

### 2.3 Degradation of PeCB

To investigate the reactivity of JS100, NZVI/BF, or JS100/NZVI/BF for the degradation of PeCB, batch experiments were conducted in 60 ml serum bottles, capped with Teflon septa, and filled with 10 ml liquid carbon-free basal medium (0.7 g of KH_2_PO_4_, 0.7 g of K_2_HPO_4_, 0.7 g of MgSO_4_·7H_2_O, 1.0 g of NH_4_NO_3_, 0.005 g of NaCl, 0.002 g of FeSO_4_·7H_2_O, 0.002 g of ZnSO_4_·7H_2_O, and 0.001 g of MnSO_4_·H_2_O per 1000 ml) and 50 µL of a PeCB stock solution (10 g L^−1^ in methanol), which resulted in an initial PeCB concentration of 50 mg L^−1^. The amount of NZVI and inoculation culture that was added to the basal medium was 0.1 g and 10^6^ cells, respectively. The NZVI/BF added contained 0.1 g NZVI. Serum bottles with basal medium and PeCB were used as controls. All cultivations were conducted at 25°C with constant agitation at 150 rpm. At given time intervals (2, 4, 6, 8, 10, 12, 16, 20, 24, 30, 36, 42 and 48 h), aqueous samples were extracted with *n*-hexane. All experimental points were triplicated with standard deviations ≤5%. All samples were filtered through a 0.45 μm filter film prior to analysis.

### 2.4 Analytical methods

#### 2.4.1 PeCB analysis

PeCB and degradation products were monitored using a Shimadzu QP2010 Ultra GC-MS equipped with a J&W HP-5 column (30 m, 0.32 mm i.d., 0.25 μm film thickness). Liquid samples (1 μL) were injected with a split ratio of 10:1. The oven temperature program was as follows: 100°C for 1 min, followed by an increase to 250°C at 25°C·min^−1^, which was then held for 0.5 min. The carrier gas was helium (2 ml min^−1^). The inlet and detector were maintained at 250°C. Concentrations were determined using standards set up in identical bottles with the same volumes of medium as in test bottles that were analyzed in the same way as the test bottles.

#### 2.4.2 Determination of hydroxyl radicals

The free radicals were trapped by a Bruker ESR 300 spectrometer at room temperature during the reaction of NZVI and O_2_. The trapping agent was DMPO (100 mM). The free radicals were verified to be •OH by DMSO, which reacts with •OH to form the methyl radical (•CH_3_). Typical spectrometer parameters were as follows: scan range, 100 G; center field, 3511 G; time constant, 300 m; scan time, 100 s; modulation amplitude, 1 G; modulation frequency, 100 kHz; receiver gain, 1.25 × 105; and microwave power, 20 mW.

#### 2.4.3 Bacterial count and morphological observation

The number of JS100 was determined by direct cell counting on a Petrof-Hausser chamber using a Zeiss Axio Imager microscope. The morphology of bacteria was observed by FE-SEM. The sample was prepared according to the methods described by Liang et al. (2013) and Xu et al. (2016) and observed after vacuum freeze drying.

## 3 Results and discussion

### 3.1 Characterization of biofilters and NZVI/BF

**FIGURE 1:**
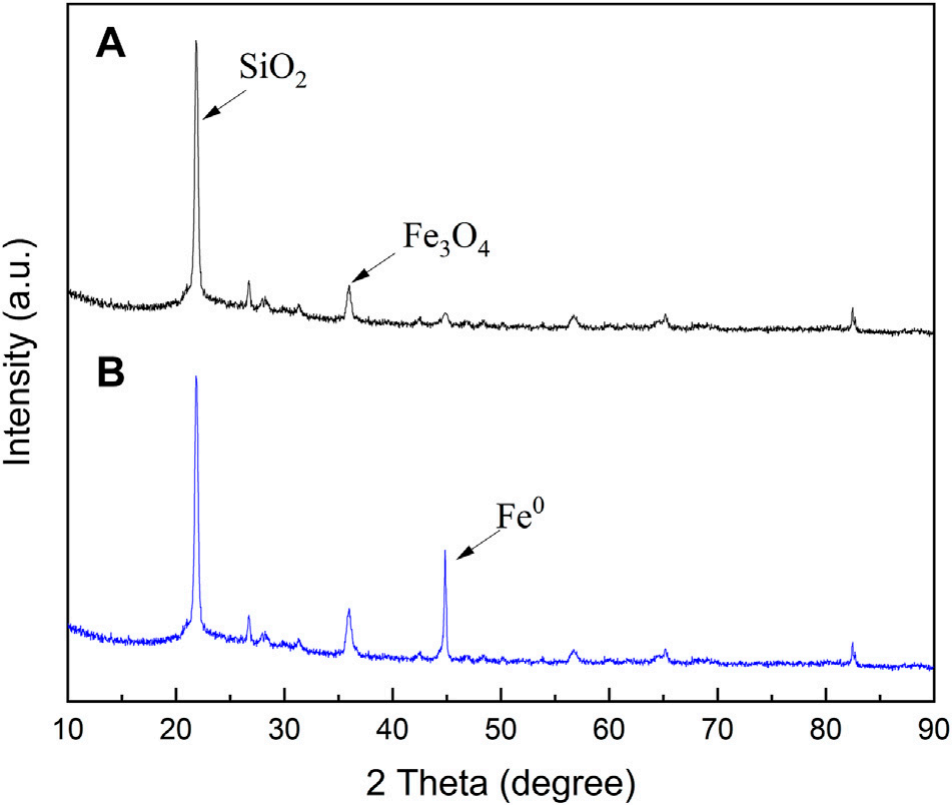
X-ray diffraction patterns of biofilters **(A)** and NZVI/BF **(B)**.

The XRD patterns of biofilters and NZVI/BF are displayed in Figure 1. Figure 1A shows a diffraction peak at 22.8° related to the high content of silicon in biofilters. A diffraction peak at 44.6° associated with the diffraction line of body-centered cubic Fe^0^ (JCPDS No. 06-0696) was depicted in Figure 1B, which suggested that NZVI particles were successfully loaded onto the biofilters. The diffractogram of NZVI/BF also showed a higher intensity peak located at 35.46° than that of biofilters, which could be related to the formation of magnetite, possibly due to the oxidation of Fe^0^ in the preparation of NZVI/BF.

**FIGURE 2:**
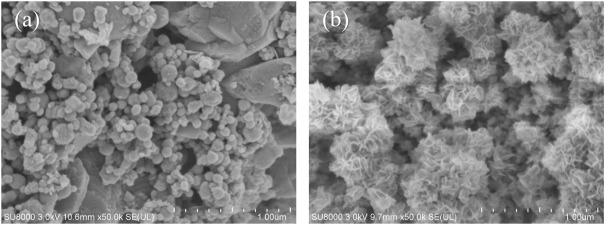
SEM images of NZVI/BF **(A)** before the reaction and NZVI/BF **(B)** after the reaction.

The SEM images of NZVI/BF are shown in Figure 2. After being loaded onto the pore surface of biofilters, NZVI retained the spherical morphology with a smooth surface and a particle size of 50–100 nm. Although it has been reported that NZVI particles formed chain-like aggregates because of their magnetic interaction (Chen et al., 2018), as shown in Figure 2A, there were few aggregated NZVI particles on biofilters before the reaction. Figure 2B shows the SEM image of NZVI/BF after the reaction. There were many flaky materials instead of spherical particles, which was consistent with the results reported by Zhu et al. (2009) and indicated NZVI had reacted with PeCB.

**FIGURE 3:**
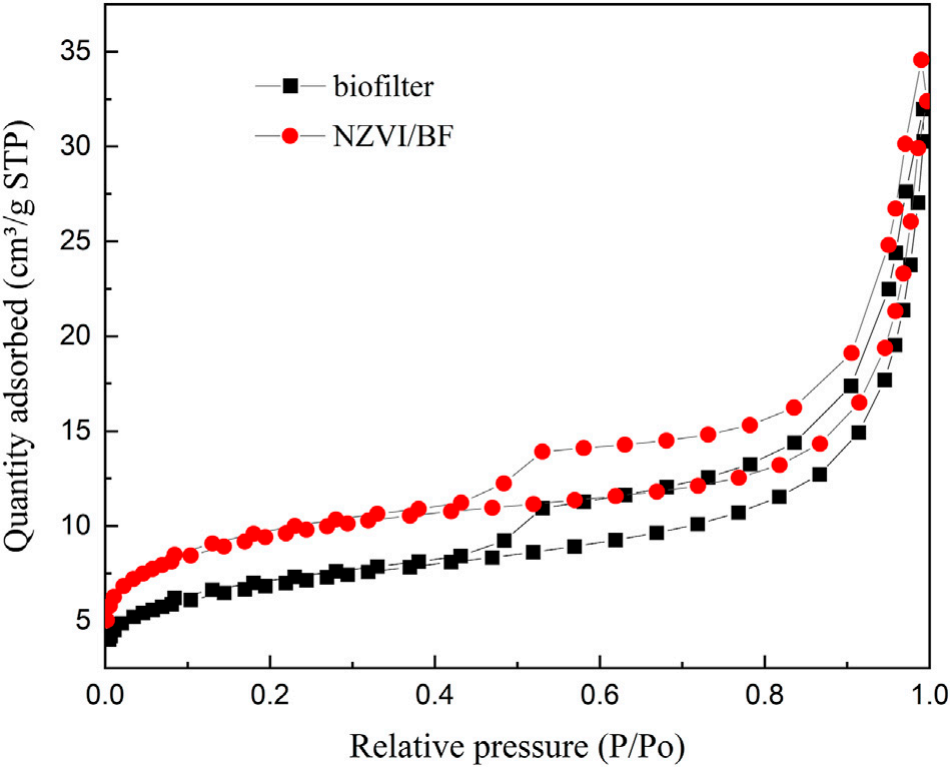
Brunauer-Emmett-Teller analysis of the biofilter and NZVI/BF.

The nitrogen adsorption-desorption isotherms of the biofilter and NZVI/BF are displayed in Figure 3, and the detailed textural data are listed in Table 1. NZVI/BF showed a BET surface area value equal to 34.5 m^2^ g^−1^, which was greater than the value described by the biofilter (25.0 m^2^ g^−1^). As shown in Table 1, the micropore surface area increased in the NZVI/BF compared with the biofilter sample. According to Figure 3 and the results of Table 1, the biofilter and NZVI/BF both had a porous network including mesopores. Although NZVI loaded on the biofilter occupied some surface area of holes, scattered NZVI particles improved the specific surface area of the NZVI/BF.

**TABLE 1:** Textural characteristics of biofilter and NZVI/BF samples.

Sample	S_BET_ (m^2^ g^−1^)^a^	S_EXT_ (m^2^ g^−1^)^b^	S_MP_ (m^2^ g^−1^)^c^	V_MP_ (cm^3^ g^−1^)^d^
Biofilter	25.0	16.6	8.4	0.0033
NZVI/BF	34.5	21.4	13.1	0.0052

^a^ Specific surface area from the BET method.

^b^ External surface area, micropore.

^c^ External surface area, micropore.

^d^ External surface area, micropore.

### 3.2 PeCB degradation and reaction kinetics

**FIGURE 4:**
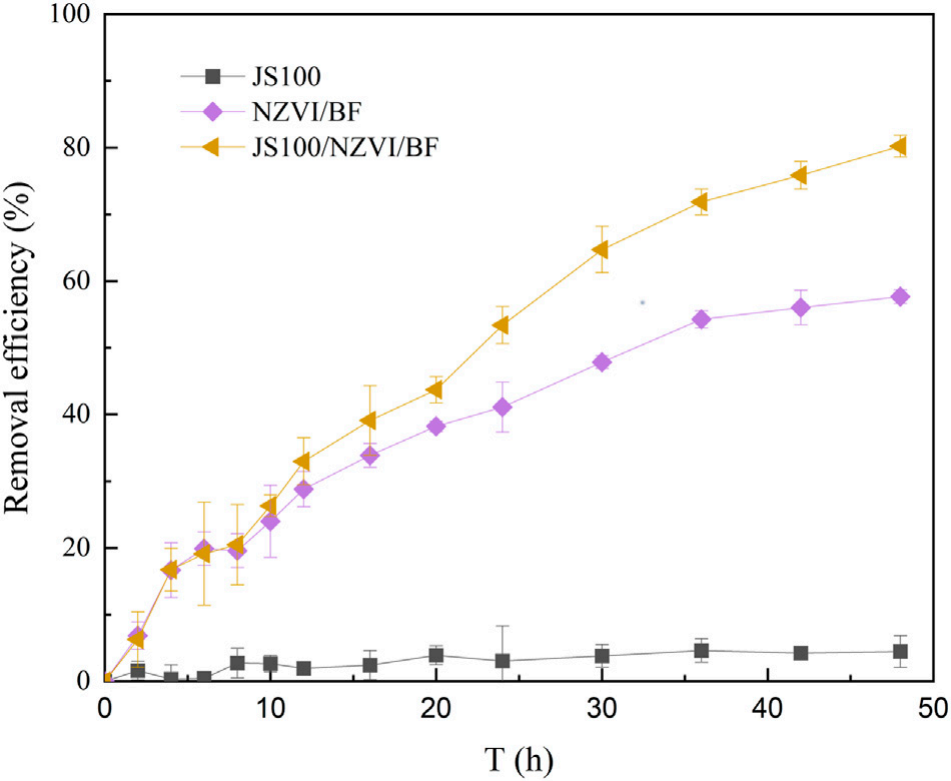
Removal efficiency of PeCB by JS100, NZVI/BF, and JS100/NZVI/BF.

In this study, the removal efficiency of PeCB by JS100, NZVI/BF, and JS100/NZVI/BF was tested. Figure 4 depicts the evolution of the removal efficiency of PeCB versus the reaction time.

*Pseudomonas* sp. JS100 obviously had no ability to degrade PeCB and could not use PeCB as carbon source; therefore, the concentration of PeCB in the solution remained at the initial level 48 h later. The NZVI/BF achieved about 57.7% PeCB conversion *via* redox reaction and adsorption. Notably, the removal efficiency of PeCB by JS100/NZVI/BF reached about 80.2% after 48 h of reaction, which indicated *Pseudomonas* sp. JS100 participated in the degradation reaction by utilizing lower chlorobenzenes produced by NZVI. JS100/NZVI/BF showed the highest degradation activity, which was closely related to the synergistic effects of scattered NZVI particles and bacteria.

**FIGURE 5:**
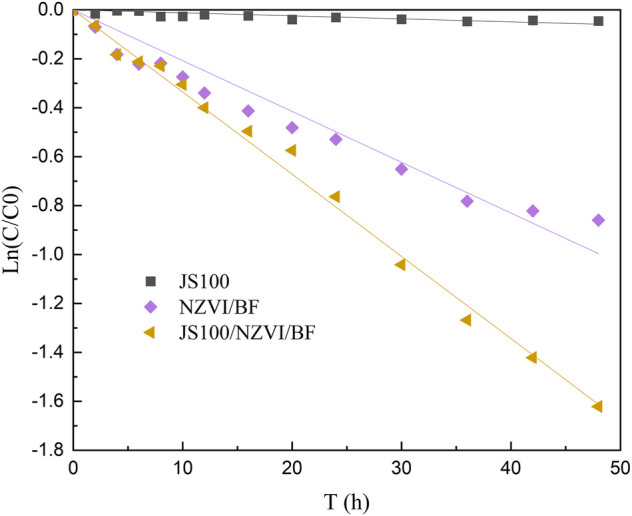
Degradation dynamics models of pentachlorobenzene.

The reactivity of JS100, NZVI/BF, and JS100/NZVI/BF was investigated through batch PeCB degradation tests, as shown in Figure 5. The entire PeCB degradation process clearly followed pseudo-first-order reaction kinetics (Cao et al., 2011):

$$\frac{dC}{dt}=-k_{obs}{C={-k}_{SA}\alpha }_{s}\rho_{m}C$$ (1)

where *C* is the aqueous phase PeCB concentration (mg·L^−1^) at time *T* (h), *k*_obs_ is the observed pseudo-first order rate constant (h^−1^), *k*_SA_ is the surface-area-based rate constant (L h^−1^ m^−2^), *α*_s_ is the specific surface area of the nanoparticles (m^2^·g^−1^), and *ρ*_m_ is the mass concentration of the nanoparticles (g·L^−1^).

The degradation rate of PeCB by JS100/NZVI/BF was significantly higher than that by NZVI/BF. By fitting the experimental data to Eq. 1, the reaction rate constants of NZVI/BF and JS100/NZVI/BF were determined to be 0.0208 and 0.0336 h^−1^, respectively. Additionally, the coefficients of determination (*R* ^2^) were all greater than 0.97, which indicated that ln (C/C0) and the reaction time had a good linear relationship.

There were several reasons for the higher efficiency of JS100/NZVI/BF. Specifically, the porous network developed in the biofilter enhanced the adsorption of the target molecules. Additionally, the biofilter acts as a carrier that reduces the aggregation of NZVI and enhances the dichlorination reaction. Finally, bacteria can utilize lower chlorobenzenes produced by NZVI to maintain all kinds of complex life activities, which can reduce the consumption of NZVI and facilitate the removal of contaminants.

### 3.3 Proposed degradation mechanism of PeCB by JS100/NZVI/BF

Previous studies showed that NZVI oxidizes organic compounds when it is used in the presence of oxygen (Oonnittan et al., 2010). NZVI reacted with dissolved oxygen, which was continuously activated, while Fe^0^ was oxidized into Fe^2+^, and a lot of H_2_O_2_ was produced on the surface of NZVI. Not only did H_2_O_2_ promote the oxidation of Fe^0^, but it also reacted with Fe^2+^ to produce hydroxyl radicals (•OH). As a strong oxidant, •OH played the main role in the degradation of organic contaminants. It has been reported that •OH can change the electron cloud density to improve the benzene ring activity so that the C-Cl bond will be attacked easily (Xia et al., 2015). As a result, higher chlorobenzenes are degraded to produce lower chlorobenzenes because of the removal or substitution of Cl^−^. Lower chlorobenzenes may continue to be attacked by •OH, resulting in their oxidization to open the loop and generate low molecular organic acids, which are gradually mineralized into CO_2_ and H_2_O.

**FIGURE 6:**
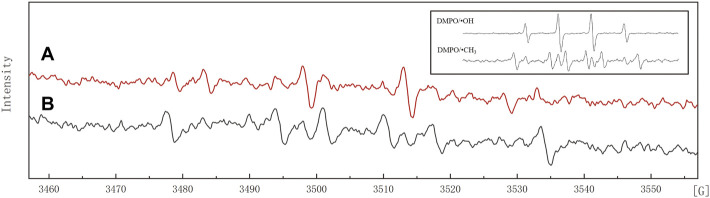
Electron spin resonance spectra. **(A)** 100 mM DMPO; **(B)** 100 mM DMPO +4% DMSO; Insert: the standard electron spin resonance spectra of DMPO/•OH and DMPO/•CH_3_.

Figure 6 shows the electron spin resonance spectra of JS100/NZVI/BF samples and the standard electron spin resonance spectra of DMPO/•OH and DMPO/•CH_3_. When incubated with the spin trapping agent DMPO, NZVI produced the DMPO/•OH adduct (Figure 6A). The formation of DMPO/•OH was markedly inhibited by the •OH scavenging agent DMSO, with the concomitant formation of characteristic DMPO spin trapping adducts with •CH_3_ (Figure 6B), which confirmed the existence of hydroxyl radicals in the process of PeCB degradation.

**FIGURE 7:**
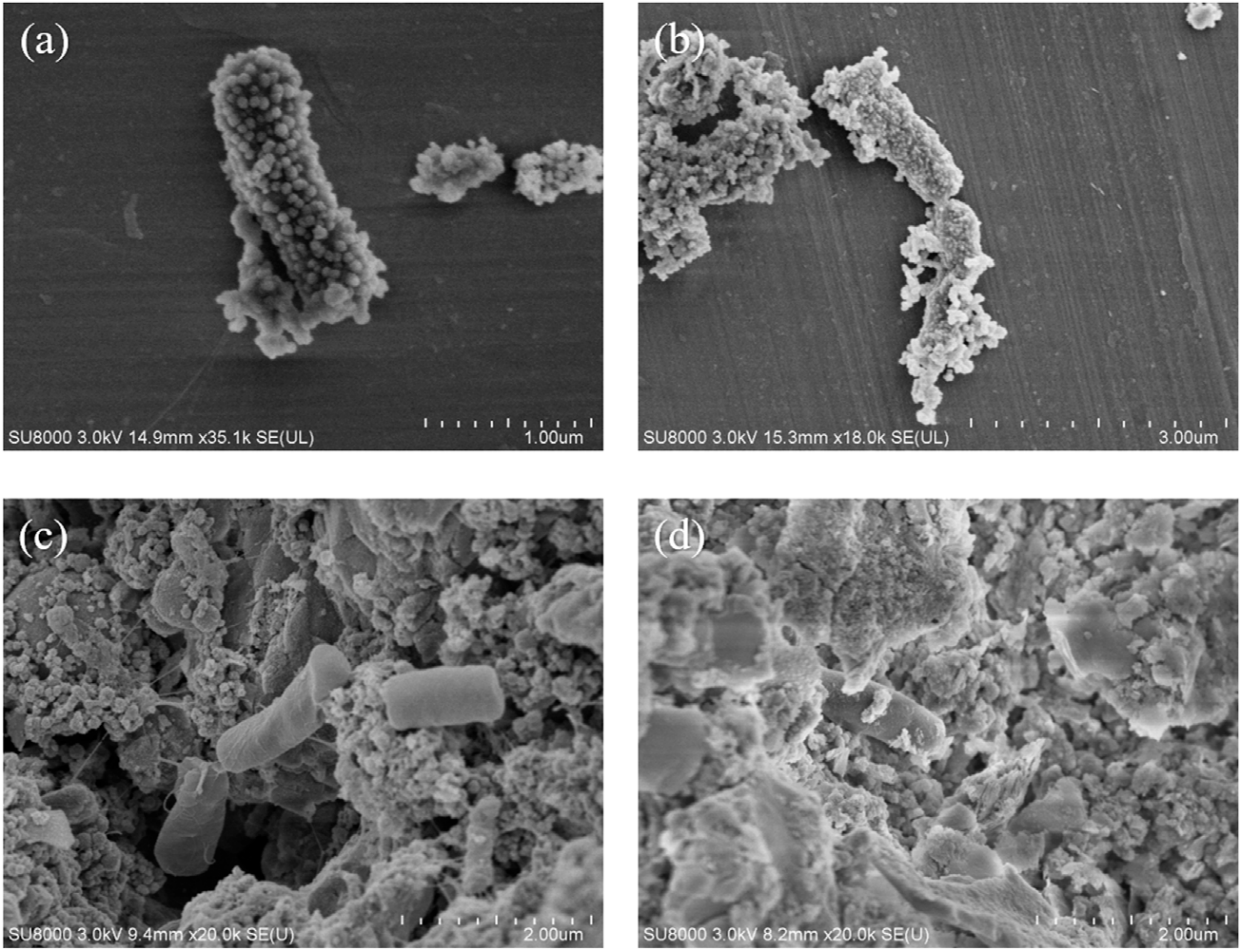
SEM images of bacteria in JS100/NZVI **(A**, **B)** and JS100/NZVI/BF **(C**, **D)**.

The SEM images of the bacteria in JS100/NZVI and JS100/NZVI/BF at 2 and 48 h are shown in Figure 7. As shown in Figures 7A,B, NZVI particles were uniformly located on the bacterial surface. The NZVI maintained obvious granular morphology except for a small portion that was oxidized, indicating that only a small amount of NZVI was involved in the reaction. However, NZVI, attaching to the bacterial surface, had reacted into some massive substance and wrapped the bacterial surface at 48 h, which would interfere with the exchange of substances between bacteria and the environment. However, as shown in Figures 7C,D, bacteria in the biofilter loaded with NZVI had no NZVI on their surfaces, retaining their original form. After 48 h, only a very small amount of the bulk oxide had adhered to the surface of the bacteria, even though the NZVI loaded onto the biofilter had been oxidized. These results indicated that biofilters provided good living conditions for bacteria in JS100/NZVI/BF. The protection of biofilters also effectively reduced the negative effects of the interactions between NZVI and bacteria, which made the material exchange of bacteria and the surrounding environment unobstructed.

**FIGURE 8:**
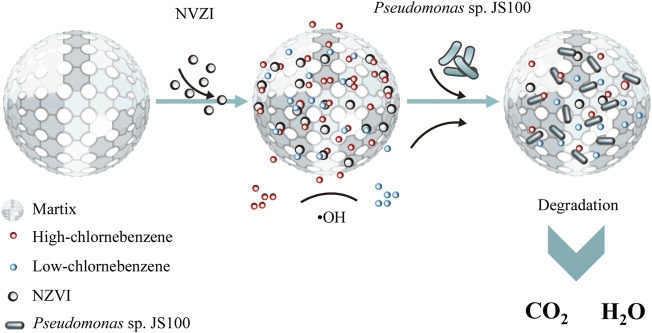
Mechanisms of dechlorination of pentachlorobenzene and synergistic biodegradation process.

The studies mentioned above indicated that NZVI played a role in the initial reaction period in the synthesis system containing NZVI and *Pseudomonas* sp. JS100 for collaborative degradation of PeCB. PeCB was attacked by •OH through an elimination reaction to form lower chlorobenzenes, which had good biodegradability. The bacteria then utilized these products as nutrients to maintain life activities and promote their growth and propagation, which reduced the consumption of •OH and facilitated the removal of PeCB (Figure 8).

In conclusion, in this study we proposed a synergistic removal strategy of PeCB. This synergistic degradation system had a higher specific surface area and better degradation ability than previous systems, enabling the realization of the full degradation effect of NZVI and *Pseudomonas* sp. JS100, which resulted in the efficient degradation of higher chlorobenzene pollutants. Moreover, biofilters imparted NZVI particles loaded on the surface with good dispersion and reactivity and provided a better living environment for the bacteria, reducing the toxic effects of NZVI. The adsorption of biofilters increases the concentration of PeCB in the pores, accelerating the degradation rate of PeCB with NZVI. Higher chlorobenzenes are then degraded to produce lower chlorobenzenes by NZVI and *Pseudomonas* sp. JS100 takes in and utilizes lower chlorobenzenes to maintain life activities. It not only improves the removal efficiency of PeCB, but also prevents the potential secondary pollution caused by the incomplete degradation of PeCB. This study provides a novel method, as one of the synthetic biology strategies (Liang et al., 2022) for the remediation of complex environments impacted by higher chlorobenzenes and other organic pollutants.

## Data Availability

The original contributions presented in the study are included in the article/supplementary material, further inquiries can be directed to the corresponding author.

## References

[B1] Barber J. L. Sweetman A. J. van Wijk D. Jones K. C. (2005). Hexachlorobenzene in the global environment: Emissions, levels, distribution, trends and processes. Sci. Total Environ. 349, 1–44. 10.1016/j.scitotenv.2005.03.014 16005495

[B2] Brahushi F. Dörfler U. Schroll R. Munch J. C. (2004). Stimulation of reductive dechlorination of hexachlorobenzene in soil by inducing the native microbial activity. Chemosphere 55, 1477–1484. 10.1016/j.chemosphere.2004.01.022 15099727

[B3] Brahushi F. Kengara F. O. Song Y. Jiang X. Munch J. C. Wang F. (2017). Fate processes of chlorobenzenes in soil and potential remediation strategies: A review. Pedosphere 27, 407–420. 10.1016/S1002-0160(17)60338-2

[B4] Cao J. Xu R. Tang H. Tang S. Cao M. (2011). Synthesis of monodispersed CMC-stabilized Fe–Cu bimetal nanoparticles for *in situ* reductive dechlorination of 1, 2, 4-trichlorobenzene. Sci. Total Environ. 409, 2336–2341. 10.1016/j.scitotenv.2011.02.045 21439609

[B5] Chen S. Bedia J. Li H. Ren L. Naluswata F. Belver C. (2018). Nanoscale zero-valent iron@mesoporous hydrated silica core-shell particles with enhanced dispersibility, transportability and degradation of chlorinated aliphatic hydrocarbons. Chem. Eng. J. 343, 619–628. 10.1016/j.cej.2018.03.011

[B6] De Bont J. A. Vorage M. J. Hartmans S. van den Tweel W. J. (1986). Microbial degradation of 1, 3-dichlorobenzene. Appl. Environ. Microbiol. 52, 677–680. 10.1128/aem.52.4.677-680.1986 3777923 PMC239096

[B7] Fang S. Huang X. Xie S. Du J. Zhu J. Wang K. (2022). Removal of chromium (VI) by a magnetic nanoscale zerovalent iron-assisted chicken manure-derived biochar: Adsorption behavior and synergetic mechanism. Front. Bioeng. Biotechnol. 10, 935525. 10.3389/fbioe.2022.935525 35875500 PMC9298784

[B8] Field J. A. Sierra-Alvarez R. (2008). Microbial degradation of chlorinated benzenes. Biodegradation 19, 463–480. 10.1007/s10532-007-9155-1 17917704

[B9] Ghasemzadeh G. Momenpour M. Omidi F. Hosseini M. R. Ahani M. Barzegari A. (2014). Applications of nanomaterials in water treatment and environmental remediation. Front. Environ. Sci. Eng. 8, 471–482. 10.1007/s11783-014-0654-0

[B10] Gunawardana B. Singhal N. Swedlund P. (2011). Degradation of chlorinated phenols by zero valent iron and bimetals of iron: A review. Environ. Eng. Res. 16, 187–203. 10.4491/eer.2011.16.4.187

[B11] He N. Li P. Zhou Y. Fan S. Ren W. (2009). Degradation of pentachlorobiphenyl by a sequential treatment using Pd coated iron and an aerobic bacterium (H1). Chemosphere 76, 1491–1497. 10.1016/j.chemosphere.2009.06.046 19596135

[B12] Liang J. Liu Y. Xie J. (2013). The improvement of bacteria sample preparation method for scanning electron microscope observation. J. Chin. Electr. Microsc. Soc. 32, 276–278. 10.3969/j.1000-6281.2013.03.016

[B13] Liang Y. Ma A. Zhuang G. (2022). Construction of environmental synthetic microbial consortia: Based on engineering and ecological principles. Front. Microbiol. 13, 829717. 10.3389/fmicb.2022.829717 35283862 PMC8905317

[B14] Oonnittan A. Isosaari P. Sillanpää M. (2010). Oxidant availability in soil and its effect on HCB removal during electrokinetic Fenton process. Sep. Purif. Technol. 76, 146–150. 10.1016/j.seppur.2010.09.034

[B15] Sarkar S. Guibal E. Quignard F. SenGupta A. K. (2012). Polymer-supported metals and metal oxide nanoparticles: Synthesis, characterization, and applications. J. Nanopart. Res. 14, 715. 10.1007/s11051-011-0715-2

[B16] Sun Y. Li X. Zhang W. Wang H. P. (2007). A method for the preparation of stable dispersion of zero-valent iron nanoparticles. Colloids Surfaces A Physicochem. Eng. Aspects 308, 60–66. 10.1016/j.colsurfa.2007.05.029

[B17] Wang Y. Fang Z. Kang Y. Tsang E. P. (2014). Immobilization and phytotoxicity of chromium in contaminated soil remediated by CMC-stabilized nZVI. J. Hazard. Mat. 275, 230–237. 10.1016/j.jhazmat.2014.04.056 24880637

[B18] Xia S. Shao M. Zhou X. Pan G. Ni Z. (2015). Ti/ZnO-MxOy composites (M = Al, Cr, Fe, Ce): Synthesis, characterization and application as highly efficient photocatalysts for hexachlorobenzene degradation. Phys. Chem. Chem. Phys. 17, 26690–26702. 10.1039/C5CP04125B 26395810

[B19] Xie Y. Dong H. Zeng G. Tang L. Jiang Z. Zhang C. (2017). The interactions between nanoscale zero-valent iron and microbes in the subsurface environment: A review. J. Hazard. Mat. 321, 390–407. 10.1016/j.jhazmat.2016.09.028 27669380

[B20] Xu X. Zhang G. Xie Z. Wang S. Zhou Y. (2016). Improvement of filter paper pack for bacteria sample preparation for scanning electron microscope observation. J. Trop. Bio. 7, 124–127. 10.15886/j.cnki.rdswxb.2016.01.021

[B21] Zhang M. He F. Zhao D. Hao X. (2011). Degradation of soil-sorbed trichloroethylene by stabilized zero valent iron nanoparticles: Effects of sorption, surfactants, and natural organic matter. Water Res. 45, 2401–2414. 10.1016/j.watres.2011.01.028 21376362

[B22] Zhang X. Wu Y. (2017). Application of coupled zero-valent iron/biochar system for degradation of chlorobenzene-contaminated groundwater. Water Sci. Technol. 75, 571–580. 10.2166/wst.2016.503 28192351

[B23] Zhou Y. Tigane T. Li X. Truu M. Truu J. Mander Ü. (2012). Dechlorination of hexachlorobenzene in treatment microcosm wetlands. Ecol. Eng. 42, 249–255. 10.1016/j.ecoleng.2012.02.017

[B24] Zhou Y. Tigane T. Li X. Truu M. Truu J. Mander Ü. (2013). Hexachlorobenzene dechlorination in constructed wetland mesocosms. Water Res. 47, 102–110. 10.1016/j.watres.2012.09.030 23089357

[B25] Zhu H. Jia Y. Wu X. Wang H. (2009). Removal of arsenite from drinking water by activated carbon supported nano zero-valent iron. Envi. Sci. 30, 1644–1648. 10.13227/j.hjkx.2009.06.046 19662844

